# What are the methodological characteristics of evidence and gap maps? A systematic review and evidence and gap map

**DOI:** 10.1002/cesm.12096

**Published:** 2024-08-05

**Authors:** Mary Fredlund, Morwenna Rogers, Noreen Orr, Dylan Kneale, Kate Allen, Jo Thompson Coon

**Affiliations:** ^1^ NIHR Applied Research Collaboration South West Peninsula, University of Exeter Medical School University of Exeter Exeter Devon UK; ^2^ University College London London UK

## Abstract

**Introduction:**

Clarity on the characteristics of methods used to produce evidence and gap maps (EGMs) will highlight areas where method development is needed to ensure these increasingly produced tools are made following best practice to assure their quality and utility. This paper aims to describe the range, nature and variability of key methodological characteristics of studies publishing EGMs.

**Methods:**

We followed a protocol, written a‐prior and informed by PRISMA and MECCIR guidelines for undertaking systematic reviews. We searched nine data bases, from 2010, for studies across any discipline that included details of their methods used to produce an EGM. Search results were screened by two reviewers independently and the subsequent data was extracted and managed according to predefined criteria. We mapped these together with the year of publication and the area of research as the two primary dimensions. We followed established methods for mapping the evidence, including the process of developing the map framework and the filters for our interactive map. We sought input and involvement from stakeholders during this process.

**Results:**

We found 145 studies from nine distinct research areas, with health research accounting for 67%. There were 11 map designs found, of these bubble plots were the most common design, before 2019, since then it has been a matrix map design. Stakeholders were involved in 47.7% of studies, 48.35% of studies stated finding gaps was an aim of their work, 42% reported publishing or registering a protocol and only 9.39% of studies mentioned a plan to update their evidence maps/EGMs.

**Discussion/Conclusion:**

Key areas of methodological development relate to: the involvement of stakeholders, the conceptualization of gaps and the practices for updating maps. The issues of ambiguity in terminology, the flexibility of visualizations of the data and the lack of reporting detail were other aspects that needs further consideration in studies producing an EGM.

## 1 BACKGROUND

Evidence and gap maps (EGMs) are one of many emerging visualization products arising from a relatively new evidence synthesis process, evidence mapping [1, 2]. Early examples include descriptive mapping studies in health [3, 4] and educational research [5, 6]. Maike‐Lye et al. identified 10 studies by 2010 [7]; in the same year the international initiative for impact evaluation (3ie) labeled the first evidence map as an “Evidence Gap Map” [1, 5, 8]. In recent years studies making EGMs are being increasingly used to collate and organize research on a particular topic, sector, subsector or domain across a range of disciplines.

A key problem with EGMs is that they are not a clearly distinct evidence synthesis product. Like other evidence mapping or scoping reviews they have a broad focus and use a systematic search to find existing research available in an area and can be a foundation for more focused syntheses [2, 4, 5, 7, 8, 9]. Their distinction seems to be related to the evidence base being displayed in a user friendly visualization [1, 8] to support evidence informed decision making and the early involvement of their target audience (stakeholders) [10].

The qualities of being freely accessible, user‐friendly and interactive, offers the prospect of a broader range of stakeholders using the EGMs made which highlights the need for those stakeholders to be involved in their design and production. In a health context this includes patients, their families, public, clinicians, researchers, and commissioners.

EGMs are made in studies across multiple disciplines meaning they are likely to be highly heterogeneous, for example, in terms of target audience, visualization form, software used and how they identify gaps. There is a need to describe the key characteristics of EGM studies, to explore their distinctiveness and highlight areas needing methodological development. For example, since stakeholder consultation is an important facet of producing a map [10], there is a need to review how this happens and areas where further clarity would be helpful.

In this article, we present an interactive EGM of key methodological characteristics of published EGMs. The overarching research question is: “what are the characteristics of the methods used to produce, present and update EGMs which impact the utility and usability of EGMs for stakeholders and/or end users?” The analysis was directed by a number of sub‐questions set out below.

a) What is the range and type of visualization approaches that have been used in published EGMs?
b) What are the available platforms for constructing EGMs and how have they developed over time to offer a range of visualization tools?
c) What is the nature of stakeholder involvement in the development of EGMs?
d) How are stakeholders included in the decisions that determine the format and content of the map?
e) How have ‘gaps’ been synthesize and displayed?
f) When and how are existing EGMs updated?

## 2 METHODS

We registered a detailed protocol, here [11]. Our systematic review and EGM have been conducted and reported according to PRISMA [12, 13, 14] and MECCIR guidelines [15, 16, 17].

### 2.1 Stakeholder involvement

We involved stakeholders from different backgrounds during this study, including researchers and academics with and without experience of visual evidence maps (*n* = 3), professionals (commissioners) in local service provision with experience of evidence mapping (*n* = 2) and members of the public with a range of patient and public involvement and engagement (PPIE) experience (*n* = 3).

We met with individuals and/or groups of stakeholders rather than convening a whole group meeting. This approach allowed for targeted conversations that related more closely to the experience and background of the wide range of stakeholders involved. Involvement included both discrete, one‐off meetings as well as on‐going discussions.

We sought feedback on the draft protocol, the synthesis plan, the framework for the EGM, the initial results to direct further analysis, the display and visualization of the map, the focus of the discussion, and dissemination. The focus of the stakeholder work was to ensure that the findings made sense to a range of audiences. Further details of the stakeholder involvement are described under “developing the framework.” The impact of stakeholder involvement can be seen in Table 1.^a^

**Table 1:** Table summarizing the impact of stakeholder involvement in this study; based on the PIRIT reporting tool kit.

Involvement activity type.	Stakeholder contribution: *Focus of the contact* and a summary of issues raised, views shared and suggestions made.	Impact: Summary of how this contribution resulted in a change or action. What influence has it had.
One‐to‐one meeting (in person)	*Study protocol*: Some suggested changes in phasing and where more detail was needed were given. More generally it was noted that it was hard to give feedback on the protocol due to lack of specific knowledge about the process.	Changes in phrasing and detail were incorporated. The views were recognized and were addressed were possible. A lay summary of the methods was developed.
Email	*Study protocol & lay summary of methods*: It was noted that things needed to be further simplified to be understood by lay stakeholder. It was suggested that there needed to be training on what the methods were for lay stakeholders to understand the processes happening or just focus the involvement activity to a small area.	Different subheadings were used and details expanded on. In terms of the lay summary it was accepted that this was not a helpful way to explain the methods and more consideration of how to do this was needed.
One‐to‐one meeting (online)	*Evidence mapping (EM), different visualizations and the map framework*: That the tabular or matrix visualizations seemed clearer than some others and that the map framework proposed in the protocol seemed appropriate. Would be interested in the final map.	It was reassuring that the direction of the research and the results would be interesting and relevant
Email	*Evidence mapping (EM), different visualizations and the map framework*: That it would be helpful to have things explained and taken through things more.	A meeting was arranged.
Group meeting (online)	*Evidence mapping, different visualizations and the map framework*: This raised the point that EM can mean different things to different people; especially for Nonacademic practitioners/commissioners. It was also raised that it was hard to be certain about the framework without seeing the results.	Their views about the protocol were recognize and were possible addressed.
One‐to‐one meeting (in person)	*Discussing the map framework and initial results*: Suggested that the choice of framework seemed appropriate but that it was hard to really undertake how it would be used by non‐professionals or the public as the topic was about methods of maps.	That the audience for this map might not be the general public but those working in the field of evidence synthesis methods.
One‐to‐one meeting (in person)	*The map framework and initial results*: That the map looked inviting but it was hard to really make sense of the detail within it and it was helpful to be walked through the map and what it contained.	That making the map framework clearer might be of benefit. This contributed to the motivation to change the map framework.
Group Meeting (online)	*The map framework and initial results*: That the map looked difficult to understand and clear guidance was needed to understand it. That is was possible that busy professionals might not explore it if its purpose to their work was not clearly obvious.	That there was a need to think about how the results were presented in the map. Map framework was reconsidered after this.

*Note*: See Newman et al. [18].

### 2.2 Eligibility criteria

We included studies describing the development or production of a visual presentation of the evidence base labeled or referred to as an EGM. A “visual presentation” referred to an image, picture, figure or diagram, chart or illustration (interactive or otherwise) from any research discipline that met one of the following definitions:

- A visual tool for presenting the state of evidence in particular thematic areas, with the aim to provide easy access to the best available evidence and highlight knowledge gaps [7].
- “A systematic [visual] presentation of the availability of relevant evidence [of effects] for a particular policy domain” where that evidence “is identified by a search following a pre‐specified, published search protocol” [5].

Sources and documented material without methodological details, for example, feature articles, opinion pieces and letters were excluded. Uncompleted studies were excluded. Other sources and publications, including protocols, working papers, and articles in conference proceedings, abstracts, editorials, dissertations, websites and chapters in textbooks were excluded. Studies published before 2010 were excluded because the first known example of a published EGM was in 2011. Studies where the “visual presentation” only consisted of a geographical map were excluded.

### 2.3 Search methods and sources

We searched nine electronic databases: MEDLINE, EMBASE and PsycINFO (via Ovid), CINAHL (via EBSCOhost), Epistemonikos, ASSIA, Social Policy and Practice (via Ovid), Web of Science Core Collection (including social care and conference abstract databases) and ProQuest Theses & Dissertations. No date restrictions were applied. Searches were conducted July 2020, and updated July 2021.

**Figure 1:**
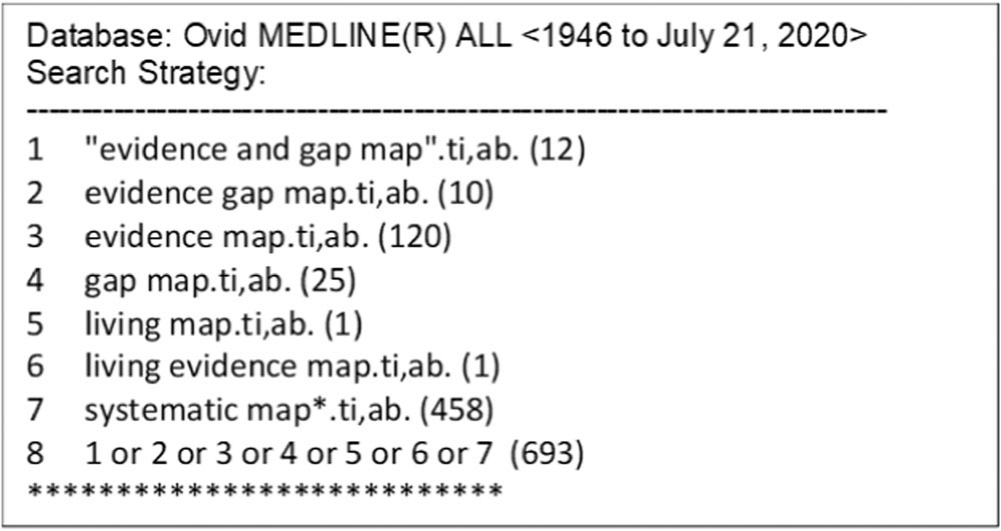
The search strategy for this review as formulated for MEDLINE.

We searched title and abstract fields for the terms: “evidence and gap map,” “evidence gap map,” “evidence map,” “gap map,” “living map,” “living evidence map,” and “systematic map*.” The search strategy is presented in Figure 1 for Ovid MEDLINE. It was adjusted for the other databases. Additionally, we searched Google scholar using the phrase ‘evidence gap map’ and reviewed the first 20 pages. We also conducted a search of gray literature via the websites of relevant organizations known to commission or produce EGMs including as 3ie, EPPI‐ Centre, and the Campbell Collaboration.

### 2.4 Screening

All records retrieved from the databases were exported into EndNote X9 (Clarivate) [19] for screening. Screening guidelines were developed in the form of questionnaires (see Appendix A). Duplicates were removed, then two reviewers (MF and KA) independently screened all study titles and abstracts according to the eligibility criteria. Following this, the full texts of remaining records were also double screened independently. Discrepancies were resolved by discussion and when needed involved the wider research team.

### 2.5 Data extraction and management

Data extraction was undertaken in EPPI‐Reviewer 4 [20]. A data extraction tool was drafted, tested and modified following piloting (Appendix B). Data extraction was undertaken by one reviewer (MF) and independently checked by a second member of the research team (KA). Any inconsistencies identified were rechecked and resolved through discussion and where necessary by consultation with the wider team.

We collected data from each study, methodological and reporting characteristics including: year of publication; research domain; availability of published protocol; methodological approach reported; methods of visualization (visualization design, format, and nature of interactivity); software used for visualization or data collation; map interactivity; the nature of stakeholder involvement (if it was reported, activities mentioned, a count of different activities, impact of stakeholder involvement on map format and/or content); the methods for identification of gaps; the aim(s) of finding gaps; the reporting of gaps; the plan to update maps and if the map was a “living” map.

To our knowledge, there is no tool for assessing the nature of stakeholder involvement.

Due to the quality of reporting it was not possible to assess the nature of stakeholder involvement in a comprehensive way. We developed a framework for indirectly assessing whether stakeholder activities impacted a map's format and content. We considered whether activities were reported, and how they were reported in the studies to make judgements about the impact of reported activities.

### 2.6 Methods for mapping

The data on each study were visualized in an interactive map generated using v.2.2.4 of the EPPI‐Mapper [21] powered by EPPI Reviewer [20] and created by the Digital Solution Foundry.

### 2.7 Developing the map framework

The top layer of the EGM is constructed as a two‐dimensional matrix framework consisting of rows and columns. Development of the framework was an iterative process. Initially, over four meetings, we presented a number of different visualization options to stakeholders (*n* = 6), with all three groups of stakeholders consulted. In addition, we explored various matrix formats, with different data presented in the rows and columns, and discussed these with our stakeholders. The resulting format, set out in our protocol, had rows representing different research domains and columns showing different map visualization designs.

The framework was derived from the evidence, with the expectation that it could be expanded to include other domains and designs found in further iterations of the map. It was determined that this would avoid the framework being overly large (with a list of all possible domain of research) and subsequently mainly empty, or full of uninformative gaps.

The completed EGM was reviewed and compared with other combinations of variables on the framework axes because feedback from stakeholders highlighted confusion and challenges interpreting and engaging with a complex map framework. Consequently, it was determined to change one axis (columns) and the segmenting variable(s) used from that specified in the protocol. The final map was constructed using a two‐dimensional matrix framework consisting of the following:

- *Columns*: showing the evidence by year of study publication.
- *Rows*: showing the different research domains the studies relate to (Area of research).

Within the cells, one variable (map visualization design) was segmented into five groups and displayed by different colors. The group name, color and information on which the 11 design codes these groups contain, are listed below.

- *Bubble plot design (red)*: Includes bubble plots/charts. These are a variant of scatter plots. They chart up to five dimensions of data: three primary dimensions (the “bubbles” x, y locations and its size) and two further dimensions by using colors and patterns for the bubbles [5, 22].
- *Matrix design (blue)*: Includes visualizations described as a “Matrix” and/or charting data in a two‐dimensional “framework” of rows and columns. These may contain a third dimension (the size/number of plots in a given cell) and further dimensions according to color and shade [10].
- *Heatmap design (green)*: Includes a visual described as a Heat‐map and/or charts two primary dimensions of data by color and color intensity. There are two main types of heat map, spatial, and grid [23].
- *Matrix‐like designs (purple)*: Includes visuals of two design codes [in data extraction], namely grid or table. These look similar to a matrix design but do not mention a methodological stage of “developing a framework.”
- *Less common designs (orange)*: Includes studies when the number of studies with a given design is small (X < 3); 6 in total.

### 2.8 Filters for map exploration

Map users can limit the data they explore by applying filters from the list below. Users can select any number of whole filter domains or any mixture of whole filters and/or parts of a filter. The number of parts that each filter has is shown in brackets next to the filter domain.

- Year of publication (*n* = 11)
- Area of research (*n* = 9)
- Map visualization design (*n* = 11)
- Study and/or Map features (*n* = 5)
- Visualization platforms, tools or software for interactive maps (*n* = 15)
- Areas where stakeholders were involved (*n* = 6)
- Gaps mentioned in different parts of the map reports (*n* = 5)
- Method used (*n* = 5)

## 3 RESULTS

### 3.1 Characteristics of eligible studies

**Figure 2:**
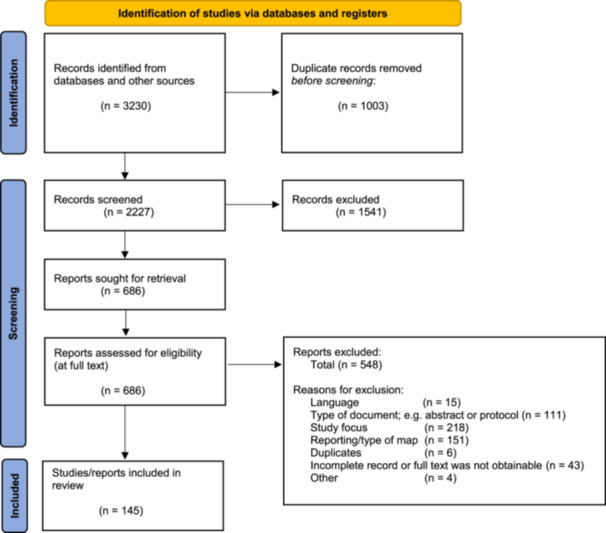
PRISMA Flow diagram of searching and screening of studies *Adjusted from:* Page et al.^24^ For more information, visit: http://www.prisma-statement.org/.

Figure 2 shows the PRISMA flow diagram summarizing the study selection process [24, 25]. Of the 3230 identified records, 2227 underwent title and abstract screening following the removal of duplicates (997). This resulted in 1541 records being excluded and left a total of 686 records which were assessed for eligibility at full text. We further removed 548 records at this stage for a number of reasons including study focus (*n* = 218; 40%), reporting reasons (*n* = 151; 28%), type of document (*n* = 111; 20%), full text was unobtainable, (43; 8%), language (*n* = 15; 3%) duplication (*n* = 6; 1%), and other (*n* = 4; 1%), e.g. incomplete record. In summary, we identified 145 eligible studies.

### 3.2 Map of included studies

**Figure 3:**
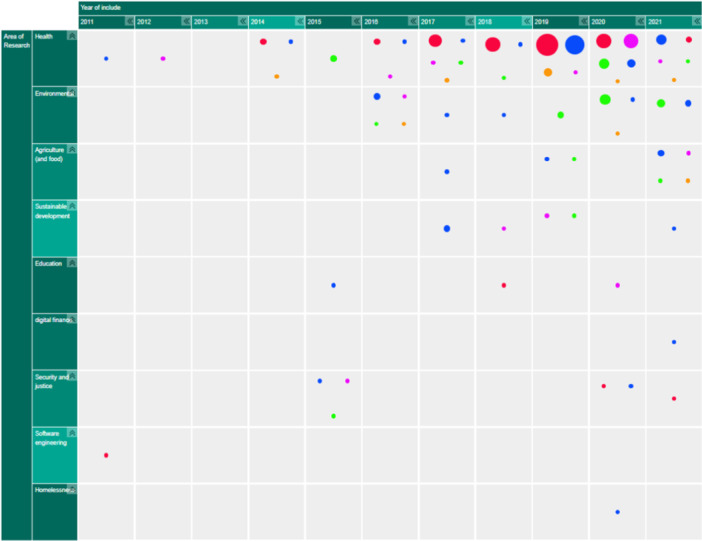
A snapshot of the top layer of the interactive evidence and gap map (EGM). Generated using v.2.2.4 of the EPPI‐Mapper (created by the Digital Solution Foundry).

Figure 3 presents snapshots of the default display of how the EGM looks and the distribution of the studies according to the two primary dimensions; “research area” (rows) and “year of publication” (columns). The interactive map can viewed following this link: https://eppi.ioe.ac.uk/cms/Portals/35/Maps/egm_characteristics.html

Each cell in the framework contains the amount of evidence that relates to that particular combination of research domain and year of publication. As already discussed, the studies in each cell are segmented into five groups of visualization designs that the studies produced, each displayed in different colors (Bubble: red; Matrix: blue; Heat map: green; Matrix‐like: purple; and other low‐frequency designs: orange).

The default display setting is mosaic where the number of squares relates to the number of studies. The user can modify the display, in the filter tab, to three other styles: “bubble‐map,” where the size of a bubble increases with the number of studies; “heat map,” where the intensity of a color displayed increases with an increasing number of studies and “donut‐map,” where the size of a ring increases with the number of studies.

The second layer of the map, accessed by clicking on a given cell, shows an overview list of all the studies relating to the cell. Each item contains details of the study title, authors, year of publication and the visualization design (indicated as a colored dot). The list order can be sorted by the user and the results grouped by visualization design.

Clicking on any study listed in this overview takes the user to the third layer of the map, the summary boxes which contain details of:

- The study title.
- A summary of key study/included map characteristics.
- A link to the full text of the report.

### 3.3 Synthesis and analysis of included studies

Studies were found in nine different research areas. Health research had by far the most maps related to it, with 97 studies (67%) in total. This was followed by Environmental research (*n* = 22, 15%), Agriculture and food (*n* = 8, 6%), International development (*n* = 6, 4%), Education (*n* = 3, 2%), and Security and justice (*n* = 6, 4%). Three other domains (Software engineering, Homelessness and Digital finance) accounted for 2%, with one study each.

**Figure 4:**
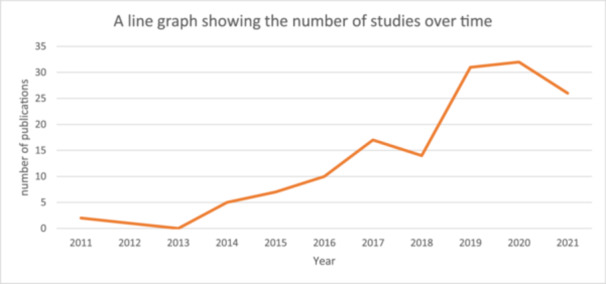
A line chart showing the change in the number of publications included in the map over time. This is a screen capture of the interactive map. The link to the map has now been added into the manuscript and the response to the peer review and can be followed to see the map.

The number of studies published each year shows fluctuations but an overall trend of an increasing number of studies over time (see Figure 4).

The terminology used to report the methodological approaches of the studies was highly variable which were synthesize into five groups with four of the five groups accounting for over 99% of studies. These were scoping review (*n* = 17), systematic review (*n* = 32), systematic map (*n* = 19), and some form of an evidence map (*n* = 97). The different ways that some form of evidence map was reported is broken down in Table 2. The other approach was an umbrella review (*n* = 1). The overall percentage is over 100 because seven studies had multiple classifications. Sixty‐two (41.6%) of the included studies reported publishing or registering a protocol. No study included a study protocol before 2015.

**Table 2:** A count of the number of studies reporting each methodological approach reported.

Methodological approach	Count	Count of breakdown for some form of evidence mapping
A systematic review	32	
A scoping review	17	
A systematic map	19	
A form of evidence mapping:	97	
Evidence mapping		69
Evidence scoping & mapping		6
Making an evidence map		12
Mapping review		5
Rapid evidence mapping		3
Evidence review (mapping)		2
Other method (umbrella review)	1	
Total	145	97

### 3.4 The range of visualizations, map designs found

A total of 11 different map designs were found which can be split into five groups. A compilation of examples of maps categorized within these five groups can be seen together in Table 3. Two of the groups, each comprising one design, accounted for 61.5% of the total. These were the Bubble chart design group (28%) and the Matrix design group (33.5%) and are shown in the map as Red and Blue respectively. Two other groups, “Matrix‐like” (containing grid and tabular designs) and the “Heatmap” design account for a further 31.1% of the total and are shown in the map as purple and green. The final group of six designs, account for 7.5% of the total maps. These visualizations designs are known as the flow diagram, the compiled bar chart, the effect direction plot, the tree map, the hierarchy list and the geographical map design. These are grouped in the map as orange.

**Table 3:** Examples of the 11 different visualization designs found.

Design group and design name	Example	Reference
**1. Main designs**		
1. A Bubble plots/charts: These are a variation of scatter plots. It charts up to five dimensions of data. Three primary dimensions (the “bubbles” x, y location and its size) and two further dimensions by using colors and patterns for the bubbles.		
	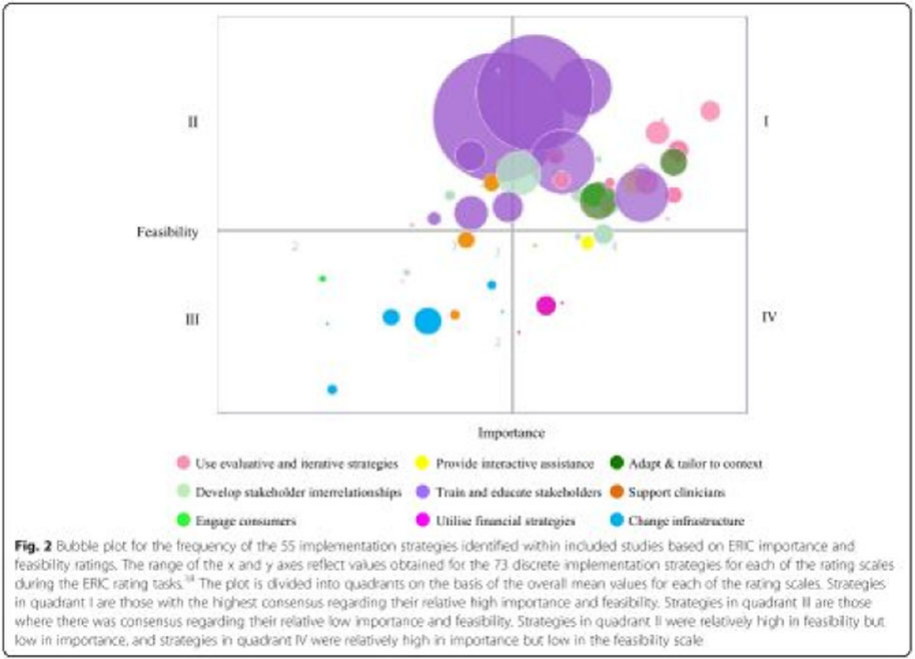	Lourida et al. [26]
	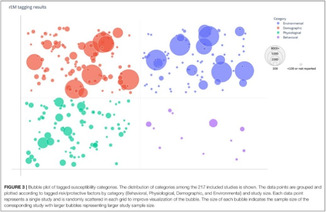	Elmore et al. [27]
	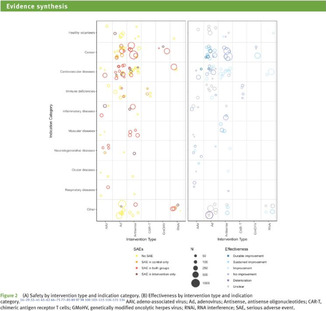	Apaydin et al. [28]
1.B Matrix: Theses chart two data dimensions in a framework (of rows and columns), a third in the size/number of plots in a given cell and further dimensions according to color and shade.		
	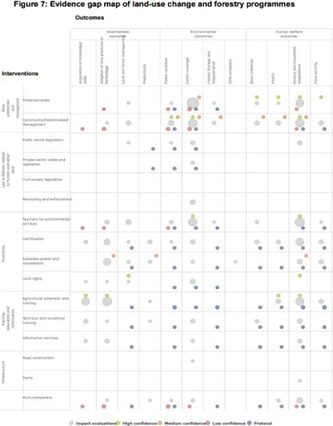	Mckinnon et al. [29]
	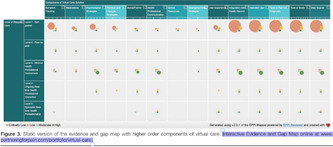	Birnie et al. [30]
	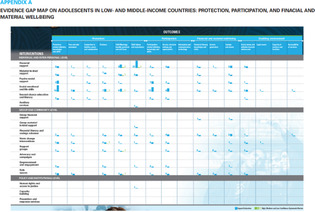	Bakrania [31]
2. Other significant designs		
2.A Tabular	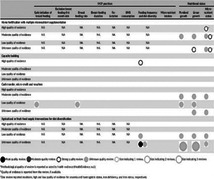	Prudhon et al. [32]
2.B Grid	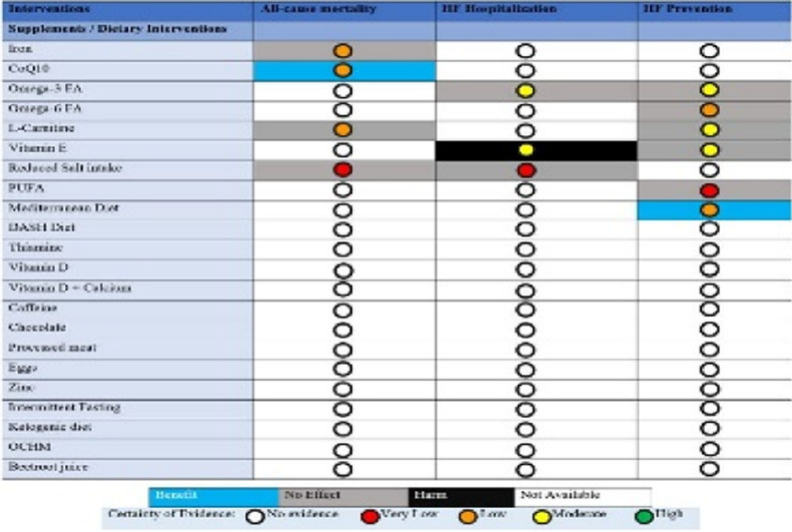	Khan et al. [33]
2.C Heat map	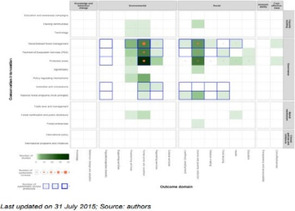 , 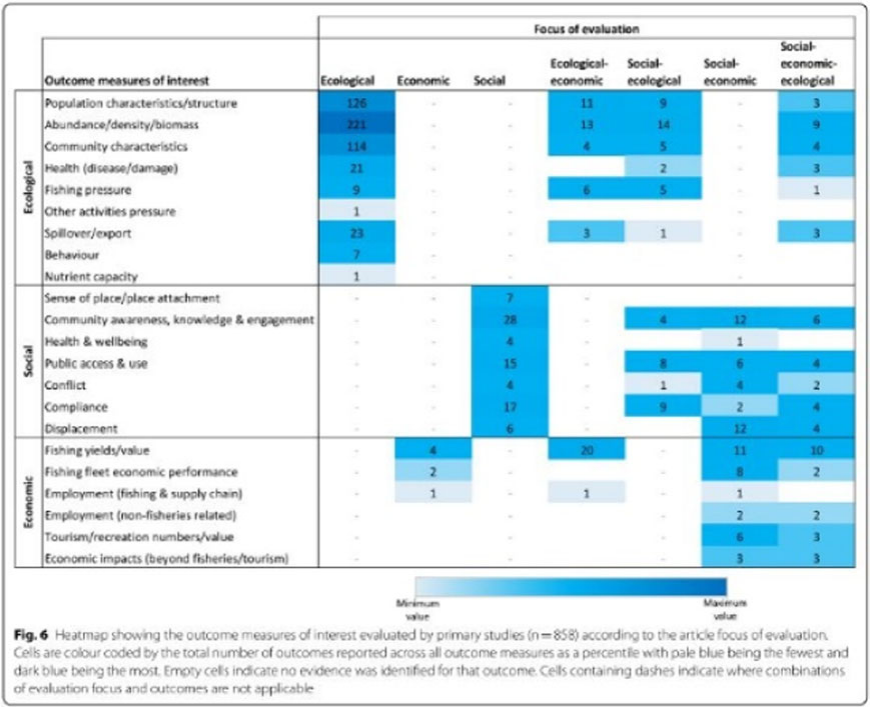	Puri et al [34], O'leary et al. [35]
3. Less frequent designs		
3.A Flow diagrams	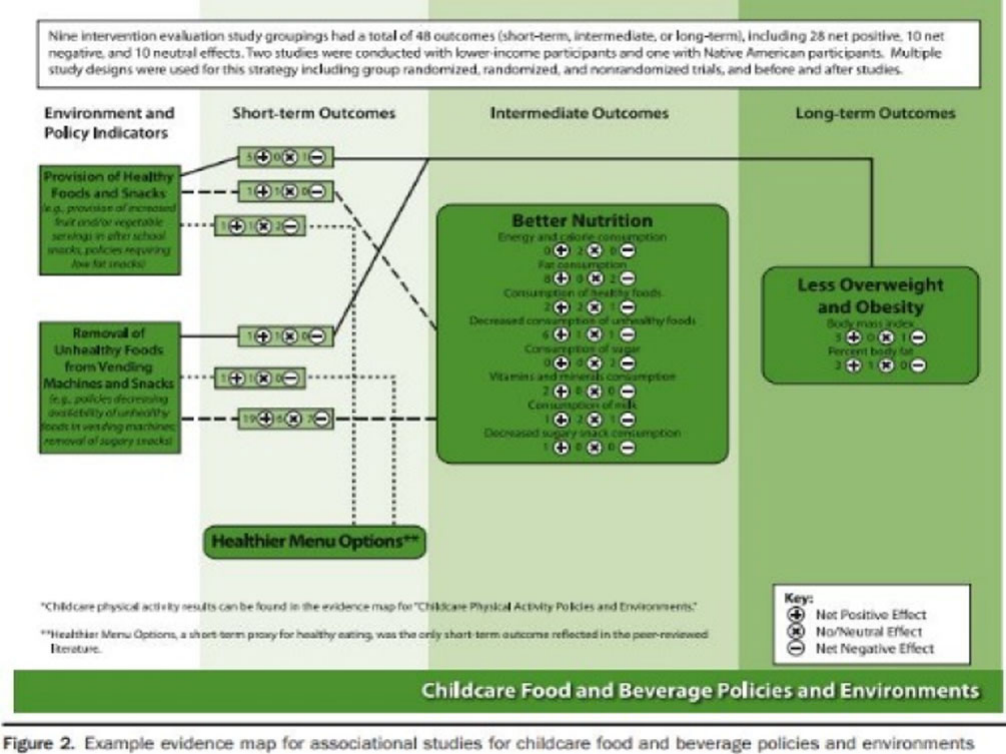	Brennan et al. [36]
3.B effect direction plot	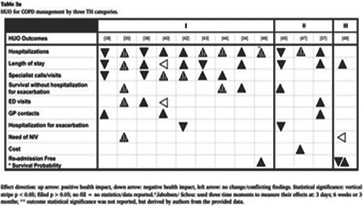	Gaveikaite et al. [37]
3.C Tree map	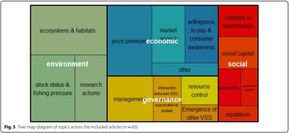	Arton et al. [38]
3.D Geographical map	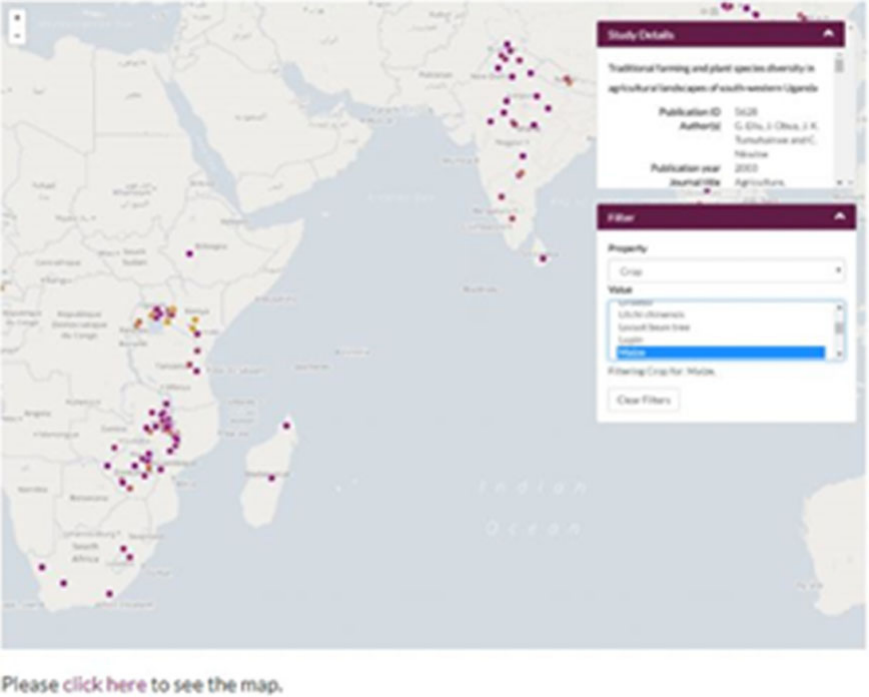	Thorn et al. [39]
3.E Complied bar chart	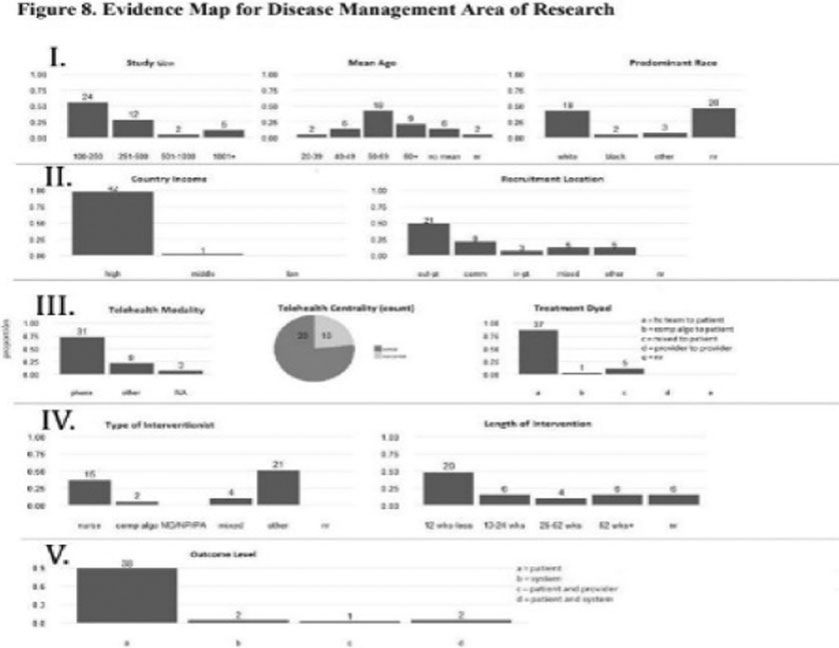	Goldstein et al. [40]
3.F Hierarchical list	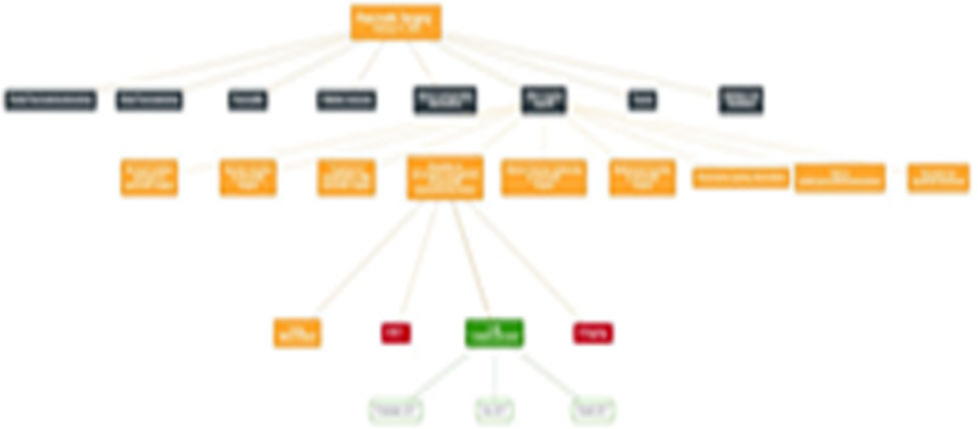	Hackert et al. [41]

### 3.5 The use and availability of platforms for constructing EGMs

Fifty (34.5%) studies involved interactive maps; the number has increased over time with most (*n* = 33) since 2019. Sixty‐six percent of these were produced using EPPI‐Mapper software [21] or were 3ie maps (*n* = 15 and 18, respectively). The remaining 17 (34%) were constructed by a number of other tools or software packages (see Table 4); for example with Evi‐atlas [42].

**Table 4:** A table showing the references, in alphabetical order, that use different tools, software or platforms to EPPI‐mapper or 3ie software to create their interactive maps.

Reference	Name of platform, tool or software mentioned*	Link to software/tool/platform (where available**)
Bakrania et al. [31]	Unclear	‐
Bakrania et al. [31]	Unclear	‐
Bouskill et al. [43]	DCIS evidence map online tool	‐
Boyd et al. [44]	Tableau and HAWC	https://www.tableau.com/en-gb; & https://hawcproject.org/resources/
Brennan et al. [36]	Transtira	www.transtria.com/evidence ***
Cheng et al. [45]	Nature and people portal	https://www.natureandpeopleevidence.org
Carslake and Dix [46]	ACER*^5^	https://documents.acer.org
Saving learning lab (2021)	Mangotree	https://mangotree.org/evidince-map ***
Eales et al. [47]	Evi‐altlas [42]	https://estech.shinyapps.io/eviatlas *^6^
CGIAR Gender Platform [48]	Tableau	https://www.tableau.com/en-gb
Hackert et al. [41]	Evi‐glance	https://www.evidencemap.surgery/
Keshava et al. [49]	Tableau & HAWC	https://www.tableau.com/en-gb
		https://hawc.epa.gov/assessment/100000047/
Saif‐Ur‐Rahman et al. [50]	Name not stated	evidencesynthesisbd.com
Schwingl et al. [51]	Tableau	https://www.tableau.com/en-gb;
Springs et al. [52]	Aero Data Lab	https://www.aerodatalab.org/
Virendrakumar et al. [53]	Unclear	‐
Thorn et al. [39]	Name not stated	https://oxlel.zoo.ox.ac.uk/resources/ecosystem-services-onfarm-conservation-map

* The name is mentioned where possible or the host of the different software.

** The link was not always available in the report/map but has been included here where it was possible to locate.

*** link no longer active.

**** HAWC + Health assessment workspace collaborative. Also see [54].

*^5^ACER Australian Council for Educational Research.

*^6^Also see: https://www.eshackathon.org/software/eviatlas.html.

### 3.6 The nature of stakeholder involvement in the development of known EGMs

Within the included studies, 75 (51.7%) reported working with stakeholders. A total of 152 different instances of involvement were counted and grouped by and displayed into six different areas (see Table 5a). The top three activities were involvement in the map framework development (*n* = 32), determining the scope of the map (*n* = 20), and developing the search strategy (*n* = 13).

**Table 5a:** A table showing the count of actvities mentioned and total studies grouped into six different areas of map making where stkeholders where invovled.

Area where stakeholders were involved	Count of activities mentioned	Total studies	Main activities named in this group
Preliminary stage(s)	44	14	Determining the scope of the map (*n* = 20) Topic selection (*n* = 9)
Map format	47	51	Map framework development (*n* = 32)
Searching	25	21	Developing the search strategy (*n* = 13) Developing the inclusion/exclusion criteria (*n* = 5)
Data processing/synthesis	6	5	‐
Dissemination activities	20	18	Reviewing the draft report (of the map) (*n* = 10), Dissemination of the findings (*n* = 9).
Not specified	9	14	‐
Total	152	‐	

There was no association of increasing involvement over time or by methodological approach used. The rate of involvement was above average in maps with the matrix design (66.7%) but less frequently reported in maps that used bubble chart, matrix‐like and heat map designs. In the matrix design the most common activity was being involved in map framework development (*n* = 23). Involvement in “bubble design” maps was mostly in the preliminary stages (*n* = 19) and reviewing the report (*n* = 6).

Due to the lack of reporting detail, it was not possible to make a detailed assessment of how the stakeholders were included in the decisions that determined the format and content of the maps. However, an assessment framework was applied which categorized the 152 instances into one of five sets, according to whether the activity could impact the map format (26%, *n* = 40), content (36%, *n* = 55) both (4%, *n* = 6), neither (18%, *n* = 27), and unclear (16%, *n* = 24) (see Table 5b).

**Table 5b:** A table of the categorized the 152 instances into one of five sets, according to whether the activity could impact the map format.

Number of activities reported per study	Count of studies	Percentage (%)
Map format	40	26
Content	55	36
Both (format and content)	6	4
Neither	27	18
Unclear	24	16
Total	71	58

Two styles of involvement were noted; discrete events such as convened workshops [55] and in a continuous fashion over the course of the research through advisory groups [56]. Three groups of stakeholders were identified which were:

i. Researchers/academics, sought for example for methodological advice.
ii. Nonacademic professionals and knowledge experts, working in the area in which the map was being produced.
iii. Those with lived experience of the subject being mapped.

The lack of detailed reporting made it difficult to accurately access the proportion from each group. Most maps indicated stakeholders were from the first two groups only and there were many instances where ‘no PPIE’ was explicitly reported; for example Saif‐Ur‐Rahman et al. [57] stated “there was is no direct involvement of patients and public in the whole process of [making the] EGM.”

### 3.7 How “gaps” have been synthesize and displayed in the EGMs identified

Analysis of the included papers revealed that there was little detail as to how gaps were constructed. This may be related to the fact that, in total, just over one‐third of studies (35%, *n* = 52) mentioned “gaps” within the overall aims of their studies. When combined with the sub‐aims/objectives, this rate increased to 48.35%, but over half of the studies (51.7%, *n* = 77) did not aim to reveal gaps in the evidence base they were mapping.

The most common place gaps were mentioned was in the 'reports' discussions, with 84% (*n* = 125) of the studies mentioning gaps. This was followed by the background/introduction sections (58%, *n* = 87), the overall aims (48.35%, *n* = 72) and the results (47%, *n* = 70). This is shown in Table 6a, which also shows that the focus on using EGMs to identify and report gaps in the evidence varies over time.

**Table 6:** a: of number and % of studies mentioning gaps by different sections of the study report by section of the report (background, aims etc.).

Area of report gaps are mentioned in	Count of studies	Percentage (%)
The background/introduction (not abstract)	87	60
The aims (anywhere)	72	49.7
The overall aims	52	36
The sub aims/objectives	27	18.6
The Methods	49	33.8
The Results	70	48.3
The discussion	125	86

Only 33% of studies (*n* = 49) mentioned or considered gaps in the method section of the reports. In most instances, it was a brief mention asserting either that it was conducting the methodological process of evidence mapping that identified the gaps [58, 59, 60, 61] or that displaying the evidence in a figure that allowed the gaps to be shown [62, 63, 64, 65, 66, 67, 68]. So there was no information about how the methodological process or visualization revealed or highlighted the gaps.

There were eight studies that had further details on how gaps were constructed in the methods, which are presented in Table 6b in three groups. The first mentioned that the identification of gaps occurred during the process of collating and reporting the results [26] or as the data was coded [69] but not how this occurred. The second group reported involving stakeholders more formally, either through a “stakeholder survey” to help prioritize evidence gaps' [70, 71], or by stakeholders scoring and ranking the identified gaps [57], or by checking the gaps make sense with the “theory of change” [72]. The third group each mentioned a process termed “gap analysis” [71, 73], or “gap map analysis” [32]. These three instances, while using the same terminology, are referring to three different processes. Only one reports detail of what the gap analysis involved. It involved surveying different groups of stakeholders to “identify where evidence [was] perceived to be sufficient or missing from the perspective of different [stakeholder groups]” [71].

### 3.8 The plans for updating maps

Within the included studies, 14 mentioned a plan to update the maps. All of these studies were published since 2018 and most (12 of the 14), since 2020. There were also seven studies that mentioned being a living map, all of which were published since 2018 and six of these also mentioned a plan to update their maps. Often these plans were aspirational [74], contingent on funding [75], limited to the lifespan of the project [76] or biannual [77].

## 4 DISCUSSION

### 4.1 Main findings

This paper focuses on key methodological characteristics of published EGMs and presents the evidence as an interactive EGM. We found *145* studies over nine different research areas, with health research accounting for 99 (67%) of these.

We found 11 visualization designs, with four accounting for 92.5% of the total. The matrix design (34%) has become increasingly common over time and surpassed the early dominance of the bubble plot design. Within our studies, 50 maps were interactive, of which 33 (67%) used a matrix design and 32 (63%) were constructed using two software packages (EPPI‐mapper and the 3ie software).

We found high variability in the language used to describe the methodological approaches employed, particularly in the way evidence mapping, which accounted for 99 (67%) of studies, was described. Just under half the authors reported involving stakeholders in the development of their EGM, 62 (42%) reported publishing or registering a protocol, only 13 (9%) mentioned a plan to update their maps and only 33% of studies (n = 49) mentioned or considered ‘gaps’ in the method section.

### 4.2 A consideration of the results

This is the first interactive EGM on methodological characteristics of EGMs of which we are aware. It reveals key methodological aspects of producing EGMs that are frequently absent or poorly reported. Clear areas of methodological need include: the inclusion of stakeholders in the process; having and reporting methods for the identification of gaps; having an update plan and an available study protocol and, for interactive maps, reporting the software or tool used to construct the maps. This is contrary to the Campbell EGM guidance [10] which presumes the use of a protocol, recommends the annual updating of maps and mentions that consultation with stakeholders is essential, albeit at the start of the process.

The limited reporting of methods for the identification of gaps may be related to the lack of any guidance on how to identify them. White et al. [10] informs that EGMs are for identifying gaps but does not go further than that. This review highlights that, as a minimum, after the map is made, a “gap‐analysis” process needs to be undertaken. Within this it is vital to involve as wide a range of stakeholders as possible, including the public in health research, to ensure that the gaps “revealed” reflect the knowledge, views and awareness of all those with a stake in the subject being mapped. There is a need to explore how this can be undertaken in practice and offer some guidance on how to manage the related challenges.

Methodological developments need to be sensitive to the presence of methodological heterogeneity or variation in character of EGMs. This difference was seen in the array of visualization designs found, map interactivity, the tools and software for constructing interactive maps, the methodological approaches undertaken and the language used to describe those approaches; particularly the way evidence mapping was described.

Heterogeneity was expected due to including studies from many research disciplines and because similar heterogeneity was found in a systematic review of the components of an evidence map [7], and noted in other recent commentaries [78, 79]. A significant source is related to the fact that the specific methodological steps taken in constructing an EGM vary with visualization design and graphical representation. Some designs contain a methodological element that is distinct from others, but each design is not fully distinct from each other. For example, the bubble plot does not need a framework development stage, like a matrix design, but a heat map may.

It is clear from this study that EGMs do not have to represent the body of evidence in a matrix design, in contrast to some recent commentaries and guidance that presupposes they do [10, 79]. However, this study reveals a trend over time, towards a matrix format. The increasing dominance of the matrix format may be a consequence of software availability, as the two prominent platforms for visualizing maps both visualize with a matrix design [10]. It may also be related to a matrix design better fitting the intention to display gaps in the evidence since this may be more challenging in a highly populated bubble plots where data bubbles can significantly overlap.

Methodological developments need not aim to eliminate the presence of heterogeneity, for example, by insisting that all EGMs only have a matrix visualization. Indeed, variation may be vital to developments in the methods for updating EGMs, for example.

### 4.3 Strengths and limitations of this study

We used an innovative approach to understanding the wealth of diversity in methodologies used to producing EGMs across various disciplines. It was based on an extensive search of the literature using many bibliographic databases and supplementary web searching. We involved stakeholders early in the design and format of the map and ensured our methods were based on available best practice guidelines [10, 12, 13, 14, 15, 16, 17, 80, 81, 82].

We had hoped to explore the diversity of software used across disciplines, identifying strengths and weaknesses of different software approaches. However, authors rarely reported the platform or software they used or discussed the rationale for their use. This information was usually determined by assessing the map or its snapshot for indications of an embedded acknowledgment in the map key.

Our map framework was derived from the literature and as such does not show all potential gaps as not all possible research domains or visualization types are represented. This was an intentional decision made to ensure that the map was not unwieldy. Following consultation with our stakeholders, we simplified the design of our map to improve accessibility. This was a deviation from our protocol and meant that we needed to group map designs to fit with software requirements. As per our protocol only publications written in the English language were considered for inclusion. We acknowledge this is a potential limitation, but believe that the impact on our findings is minimal.

### 4.4 Implications

This paper highlights a number of implications for researchers conducting EGMs. Within a given project, researchers need to consider and report clearly (in protocol and write up) why they decided on a given visual representation of the data (visualization design) and other features, like interactive platform and/or software. To report this, researchers need to be aware of things including: (i) how the evidence informs their design choice, (ii) how choices impact how the data is understood and its usability, (iii) the constructed nature of apparent gaps and (iv) to acknowledge that the perceptions of what is insufficient evidence (a gap) may vary among different stakeholders. Similarly, researchers need to consider that the same evidence base may require different visualizations and map features for different target users of the map including researchers, commissioners, knowledge professionals or those with lived experience of the area being mapped is important. However there is currently little existing research which explores how useful maps are in decision making and which features increase their usefulness, to whom.

Within the research community, holding a shared understanding that there is a suite of methods suitable for producing an EGM is helpful alongside an appreciation that the landscape of software for data visualization is changing rapidly which means the processes and methods that can be used is very much in flux. Although the factors that have and will determine the pace, direction and nature of map features are not clear from this review We feel the nature of software development is one of the greatest factors that is influencing the methods and design choices is the availability of programmes to support the effective visualization of the data. Most of the EGMs identified in this paper were produced using EPPI‐Mapper [21] which is readily available and familiar to the health research community. However, other options are available such as Evi‐atlas [42], produced through the Evidence Synthesis Hackathon collaborative.

An important area for development is supporting a map's usefulness and utility. This means supporting a user's ability to interact with and explore the data and would involve. Going beyond visually representing the evidence base to visualizing the data it contains. This can support maps being useful in other ways; e.g. conceptual development. There is also a related need for work to consider if and how stakeholders wanting different aspects of maps to developed, can influence this landscape. Ideally, innovations in map software and tools need to be linked to elements that are most valued by map users and that support them to understand, explore and utilize the data a map contains.

Involving a diverse range of stakeholders at key stages of map making should help ensure that an EGM is useful, relevant, and useable. Researching how the practices of involving stakeholders can influence a map's format and content is needed. Guidance on how and when to involve stakeholders in EGM production and how to record and report their impact would help facilitate methodological clarity in this area. This work would need to be sensitive to the different meanings that ‘stakeholder involvement’ has in different disciplines.

Producing an EGM is time consuming and costly which is likely to explain why the maps found are neither annually updated nor planned to be. This has implications for a map's usefulness for decision making, especially in instances where the body of the evidence will have changed over time. Therefore, the effective use of machine learning and AI to facilitate accurate and timely map updates is an important area of development need. There is active work and development in this case with the Eppi centre [83] at the forefront in this area with fully‐automated screening, and the process of data extraction may in time be feasible in a reliable manner. At this time only a few maps created through automated processes have been published [83] although these remain the exception. Until more widespread testing and adoption of automated processes, regular updates of maps are likely to remain the exception.

However, sustaining this will rely on infrastructure funding beyond the end of a project and the matter of a different mechanism of researcher acknowledgment, over paper authorship, for those involved in updates, so that their contributions can be acknowledged and valued within academia.

## 5 CONCLUSION

This EGM provides a comprehensive snapshot and summary of the extent and distribution of published EGMs. They are being used in a wide range of research domains with a variety of visualization designs to represent the body of evidence. It clearly highlights that EGMs need methodological development to involve stakeholders in the process of map making, updating maps and how gaps are constructed. This, together with tackling the issues of ambiguity in terminology and the availability of funding, will help provide better detail for reporting and ensure more utility for map users who need to use maps in their decision making.

## AUTHOR CONTRIBUTIONS

**Mary Fredlund**: Conceptualization; data curation; formal analysis; investigation; methodology; project administration; validation; visualization; writing—original draft; writing—review & editing. **Dylan Kneale**: Conceptualization; methodology; supervision; validation; writing—review & editing. **Kate Allen**: Data curation; investigation; validation; writing—review & editing. **Jo Thompson Coon**: Conceptualization; methodology; supervision; writing—review & editing.

## CONFLICT OF INTEREST STATEMENT

One author (Dylan Kneale) works within the Eppi centre at UCL.

## DISCLOSURE

The views expressed in this publication are those of the author(s) and not necessarily those of the National Institute for Health and Care Research or the Department of Health and Social Care.

## Supporting information

Supporting information.

Supporting information.

## Data Availability

The data that support this findings of this study are available from the corresponding author upon reasonable request.

## Footnotes

a Adapted from the PIRIT toolkit 18. Newman A BE, Seddon K, et al. Launch of the public involvement in research impact toolkitBMJ accessed at https://www.cardiff.ac.uk/marie-curie-research-centre/patient-and-public-involvement/public-involvement-in-research-impact-toolkit-pirit. Supportive & Palliative Care 2023 3:A7‐A8.
