# Supplementary material for: What are the methodological characteristics of evidence and gap maps? A systematic review and evidence and gap map
Source: Cochrane Evid Synth Methods. 2024 Aug 5;2(8):e12096. doi: 10.1002/cesm.12096 (PMC11795909; doi:10.1002/cesm.12096)
Supplement: Supplementary file 1 — Supporting information. [file CESM-2-e12096-s001.pdf]

Screening guidance for title/abstract screening of the review looking to identify and appraise the methods and approaches used in the development and production of Evidence and Gap Maps (EGM):

Aims and objectives:

This scoping review aims to identify, describe and appraise the approaches used in helping to make the evidence presented within published EGMs useable and meaningful for those whose decision making may be enhanced by access to and interaction with the available evidence base that exists around a particular issue; for example clinicians, commissioners policymakers, service users patients and their families.

Search focus:

This review will be guided by the overarching question 'what are the characteristics of the methods used to produce, present and update Evidence and Gap Maps (EGM) which impact the utility and usability of EGMs for stakeholders and/or end users'.

Key terms and their definitions:

At this point in time there is no one accepted term for an EGM. To assist in this screening the following definitions taken from key references are offered as a guide.

Evidence Mapping, systematic search of a broad field to identify gaps in knowledge and/or future research needs that presents results in a user-friendly format often a visual figure or graph, or a searchable database[1].

Evidence Gap Map/Evidence Map/EGM: There is no widely accepted definition of an EGM. Miake-Lye et al (2016) identified 5 components of a map; these being (identify gaps or needs, audience engagement/user-friendly products, broad field, systematic process, and visual depiction). Two definitions suggested for use are as follows:

- A visual tool for presenting the state of evidence in particular thematic areas, with the aim to provide easy access to the best available evidence and highlight knowledge gaps[2].

- ‘a systematic [visual] presentation of the availability of relevant evidence [of effects] for a particular policy domain’. Where that evidence ‘is identified by a search following a pre-specified, published search protocol’[3].

N.B: In this review an EGM may, but does not need to be, ‘accompanied by a descriptive report to summarize the evidence for stakeholders such as researchers, research commissioners, policy makers, and practitioners’ which is an element that Saran and White (2018)[3] consider is optional to their definition.

Inclusion criteria:

	**Inclusion**
Study methods	All study designs that describe, report, discuss the development or production, or planned development or production, of an EGM/s or the updating of an EGM/s.
Language	English only
Time Frame	Published at any time.
Main content	Contains the objective, methods, results or discussion sections of the report, the methods used or the process involved in developing, or the production of an EGM

Screening questions:

1. Does the **title or abstract** use English?
   a. Yes: continue screening
   b. No: stop screening

2. Does the **title or abstract** indicate that the study reports relates to an EGM?
   a. Yes: continue screening
   b. No: stop screening

3. Does the **title or abstract** indicate that the reference in questions describes, reports on, plans to report on (e.g. the protocol) or discusses the methods used in any part of the development, production or updating of an EGM?
   a. Yes or Unsure/Unclear: continue screening
   b. No: stop screening

Decision: **Should this article be included?**

1. **Yes**: if all 3 screening questions answered is Yes or Unsure/Unclear
2. **No**: if at least one answers definitely "No"

Screening instructions:

- Please read the title/abstract of each reference and then with reference to the inclusion/exclusion criteria and/or the screening questions in these guidelines, please indicate if you have decided that the paper/report/article should be included or excluded by using one of the following codes:
  - 0 = EXCLUSION
  - 1 = INCLUSION

- It would be helpful to keep track of the reasons why you have decided to exclude each paper, if the choice to exclude was not clear cut. Instead of providing specific codes for different reasons, please make a note of the reason in your column after indicating whether it is include/exclude (where necessary). Examples include:
- Please make a note if you have included a paper but are unsure about it in some way.
- If the paper is a duplication, please screen and then add a further code of '2'.
- If you have any questions please contact the primary screener at any time who will where needed contact the rest of the research team for advice.

Post screening procedure:

Screening results will be joined together and compared for agreement/disagreement. Any discrepancies or differences in screening score will be resolved by discussion between screeners and if needed with input from the wider research team.

Reviewer details:

Reviewer number: 1
Name: Mary Fredlund
Coding location: CUSTOM 8

Reviewer number: 2
Name: Kate Allen
Coding location: CUSTOM 7

References:

1. Miake-Lye IM, Hempel S, Shanman R, Shekelle PG: What is an evidence map? A systematic review of published evidence maps and their definitions, methods, and products. Syst Rev 2016, 5:28.
2. Snilstveit BV, Martina; Bhavsar, Ami; Gaarder, Marie: Evidence gap maps - a tool for promoting evidence-informed policy and prioritizing future research. 2013.
3. Saran, A. and White, H. (2018), Evidence and gap maps: a comparison of different approaches. Campbell Systematic Reviews, 14: 1-38. doi:10.4073/cmdp.2018.2
4. Polanin JR, Pigott TD, Espelage DL, Grotpeter JK. Best practice guidelines for abstract screening large-evidence systematic reviews and meta-analyses. Res Synth Methods. 2019;10(3):330-342. doi:10.1002/jrsm.1354

Acknowledgments:

These guidelines have been prepared with reference to Polanin et al (2019)[4] with thanks to Kate Allen for her advice and feedback.

Appendix A – screening guides: ti/ab and full text

Screening guidance for full text screening of the review looking to identify and appraise the methods and approaches used in the development and production of Evidence and Gap Maps (EGM):

Context: These guidelines are to help with the full text inclusion/exclusion screening of the search results for the scoping review into methods of visualisation for Evidence Maps.

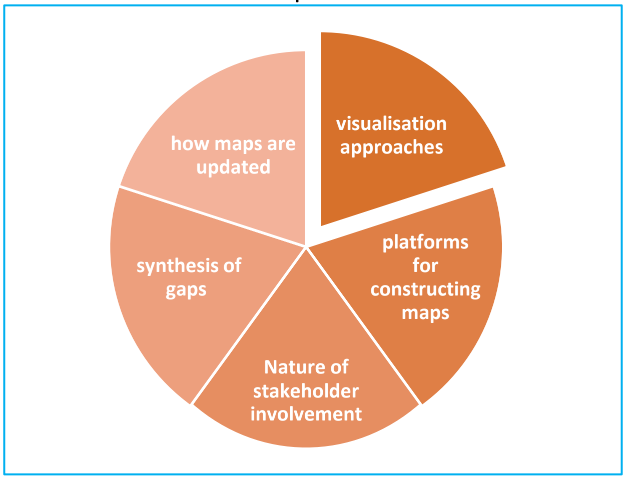

Screening directions: Please read and familiarise yourself with the exclusion criteria presented in table 2. A short summary version is found in table 1. Further details of one exclusion reason (0b) is found in table 3. Please use these in conjunction with screening orientation/training.

The review aim is to: identify, describe and appraise the approaches used in helping to make the evidence presented within published EGMs useable and meaningful for those using them (for example clinicians, commissioners policymakers, service users patients and their families).

The review research question is: 'what are the characteristics of the methods used to produce, present and update Evidence and Gap Maps (EGM) which impact the utility and usability of EGMs for stakeholders and/or end users. Specific the analysis will focus on the things in the diagram below

**Table 1: Summary of codes**			
**INCLUDE**			
	CODE	REASON	WHAT THIS RELATES TOO.
	1	An include	Reports a study that produces a visual display of the data – which synthesises three different elements.
**EXCLUDES**			
	CODE	REASON	WHAT THIS RELATES TOO.
	0a	Type of document	For example a list of conference abstracts /2 page summary
	0b	Focus/detail of the study reference	a) Relates to another type of evidence synthesis [0b]<br>b) Relates to another type of evidence mapping [0b]<br>c) Relates to the systematic nature of the methods [0b m]<br>d) Relates to the presentation of the evidence mapping undertaken &/or reported. [0b (v), (t), (v/t)]<br>e) Relates to other study focus things [0b (oth)]
	0c	Level of reporting detail	The reference is about a map- but does not have any or adequate details of the methods used to. For example a study protocol

	0d	Duplicated report	There is another copy of the same report in the library
	0e	language	Not in English
	0f	Other	For example the reference is incomplete or not available at full text

Screening instructions: Please ask about anything that is not clear and undertake the following:

- Please screen the full texts in endnote and record the reason(s) to exclude a reference in the custom 7 field using the codes given in table two.
- Please make notes detailing why you have excluded as relevant.
- If you screen a reference and determine it is not to be excluded (so an include) please note this by adding the code '1' into the custom 8 field.
- Please make any other notes or comments that you feel suitable.

Table 2: details of the exclusion criteria for the full text screening		
	**Exclusion criterion code**	**Detail of Reason to exclude**
**1**	**Exclude 0a:**<br><br>**Type of document**	**The reference is not a reporting document about a study.** So, for example, the reference is:<br>• a list of abstracts from a conference,<br>• a one/two page magazine summary of a study (may look similar to the abstract),<br>• a plain language summary<br>• an summary/executive summary<br>• the paper is a discussion of methods for Evidence synthesis<br>Example: Apaydin, E. A., et al. (2020).
**2**	**Exclude 0b:**<br><br>**Study**<br><br>**Focus/type of mapping/**<br><br>**methods**	**The reference has a different focus or does not have the required synthesises. So:**<br><br>a) The reference is not reporting on evidence synthesis (e.g primary research) [0b]<br>b) The reference is not reporting evidence mapping (so of evidence synthesis [0b]<br>c) The reference does not report having systematic methods [0b m]<br>d) The reference presents an evidence map in a descriptive form and there is no appropriate visualisation presenting the evidence available.<br>e) There is something else about the study that gives rise to it being excluded<br><br>Please see table 3 for more details and examples about these reasons.
**3**	**Exclude 0c**	**The reference is about a Systematic map or evidence (and gap) map but does not have any or adequate details of the methods used to.**

	**Detail of reporting**	This means that the document is not reporting the study methods AND findings but mentions the study in another way. **This includes study protocols.**<br><br>N.B this may have been excluded with code '0a' - 2 type of document. Ideally please code protocols 0c rather than 0a.<br><br>Example: Malhotra, S., et al. (2021).
4	**Exclude 0d:**<br><br>**Duplication**	**The reference is a duplicate of another reference.**<br><br>This means there is another document that is the same. Please add a note including the word 'duplicate' in this and the other duplicated record if possible. If the document reports the same study (that is otherwise included) please code as an include and note it is linked to another
5	**Exclude 0e:**<br><br>**Language**	**The reference is not in English.**<br><br>This refers to the body of the writing and is still excluded if there is a title or abstract in English<br><br>Example: Santaella, D. F., et al. (2020
6	**Exclude 0f:**<br><br>**Other**	**The reference is excluded for an – other reason.** This includes:<br>• The reference is not complete and important details to find the full text are missing<br>• The reference is date is prior to 2010.<br>• The reference cannot be obtained at full text.<br>• The reference has a delayed publication date – due to an embargo.<br>Example: Ziegler, B., et al. (2020).

**Table 3: Further Detail of Reason to exclude related to the focus/detail of the study**
**a) Relates to another type of evidence synthesis [0b]**<br><br>**Exclude using the code 0b when:** the reference is not reporting on evidence synthesis (e.g report primary research)
**b) Relates to another type of evidence mapping [0b]**

Exclude using the code 0b when: The reference is:

- mapping the results from primary research Example: Cruickshank, M., et al. (2021).
- mapping a concept, a framework or a theory. Example: De Almeida, L. G., et al. (2020).
- something similar. Example: Brants, H. S. and B. Ariel (2020)

c) Relates to the systematic nature of the methods [0b m]

Exclude using the code 0b (m) when: The methodology does not follow a predetermined systematic process (for example it does not have a documented search strategy or inclusion/exclusion criteria).

Ideally it would mention a (published) protocol – but PROSOERO do not register these for all evidence mapping. Therefore it is considered sufficient (and not excluded) if it mentions something similar to

- We 'defined a priori explicit inclusion criteria' [8] or
- 'We developed an explicit and exhaustive search strategy'[8] or
- 'A pre-defined set of inclusion criteria was used'[9]

AND the reference shares details of that search strategy

- In the text or in a table
- Or informs that the search strategy can be seen in an Appendix or in a supplementary file.[10]

N.B: Please also note that sometimes the protocol is not mentioned in the method but elsewhere – for example in the result section or in the small print 'notes' at the end of the document.

An example of a study that does not report enough of the methods as there is no search strategy is: Whyle, E. and J. Olivier (2020).

Where the process says is follows the guidelines of another reference - but does not in itself report enough to be sure it is 'pre-determined' and systematic then this is NOT enough and it is excluded. For example the reference might way they follow the 'guidelines of Peterson et al (2008)[12]' but does not then report what it has done in any detail.

d) **Relates to the presentation of the evidence mapping undertaken &/or reported. [0b (v), (t), (v/t)]**

Appendix A – screening guides: ti/ab and full text

**Exclude using code 0b & add (v), (t), (v/t) when:** There is no appropriate visual depiction presenting an overview of the available evidence that was mapped in any form. Regarding this please exclude when:<br><br>• The only visualisation relates to another sort of evidence synthesis. Including a<br>○ Risk-of-bias overview summary. Including 'Amstar' visuals.<br>○ Time trend of number of articles in study<br>○ Trend of study designs over time<br>• When the only type of 'visualisation' is a table presenting the results. Since this is within the remit of a 'descriptive' evidence map[13].<br>Example(s): Althuis, M. D. and D. L. Weed (2013)[14]; Welsh, E. J., et al. (2015)15<br><br>• When the only visualisation(s) do(es) not offer an overview of the evidence base. This includes:<br>○ A graph/plot or picture of an overview of the papers included (over time or location the research is from)<br>○ A picture or an atlas/map or similar<br>Example: Sopjani, L., et al. (2020)[16]; Haddaway, N. R., et al. (2017)[17]<br><br>The type of visualisation that would be included is:<br><br>• One that is displaying at least two different elements that the study has categorised or 'coded' from the evidence base they are looking at AND that they are displayed in a way that has combined them to generate a new element/component of analysis.<br><br>N.B: It is important to check that there is no link to or mention of such a visual depiction in an additional file or a mention of a link that a visual depiction is published on the internet in its own right. See also Miake-Lye et al (2016)
**e) Relates to other study focus things [0b (oth)]**<br><br>There is something else about the study that gives rise to it being excluded

REFERENCES

1. Apaydin, E. A., et al. (2020). "AN EVIDENCE MAP OF GENETIC THERAPIES." Journal of general internal medicine 35(SUPPL 1): S14-S14.
2. Malhotra, S., et al. (2021). "PROTOCOL: evidence and gap map: studies of the effectiveness of transport sector interventions in low- and middle-income countries." Campbell Systematic Reviews 17(1)
3. Santaella, D. F., et al. (2020). Evidence Map: clinical efectiveness of yoga. PNPIC: 24-24.
4. Ziegler, B., et al. (2020). "Evidence and Trends in Burn Wound Debridement: An Evidence Map." Plastic surgery (Oakville, Ont.) 28(4): 232-242.
5. Cruickshank, M., et al. (2021). "Identification and categorisation of relevant outcomes for symptomatic uncomplicated gallstone disease: in-depth analysis to inform the development of a core outcome set." BMJ open 11(6): e045568.

6. De Almeida, L. G., et al. (2020). "Data Analysis Techniques in Vehicle Communication Networks: Systematic Mapping of Literature." IEEE ACCESS 8: 199503-199512.
7. Brants, H. S. and B. Ariel (2020) "Evidence Map of School-Based Violence Prevention Programs in Israel." INTERNATIONAL CRIMINAL JUSTICE REVIEW.
8. Winters, N., et al. (2019). "Using mobile technologies to support the training of community health workers in low-income and middle-income countries: mapping the evidence." BMJ Global Health 4(4): e001421.
9. Winters, N., et al. (2017). "Physical, psychological, sexual, and systemic abuse of children with disabilities in East Africa: Mapping the evidence." PLoS ONE [Electronic Resource] 12(9): e0184541
10. Wei, H., et al. (2019). "Therapeutic effect of angelica and its compound formulas for hypertension and the complications: Evidence mapping." Phytomedicine 59: 152767.
11. Whyle, E. and J. Olivier (2020). "Social values and health systems in health policy and systems research: a mixed-method systematic review and evidence map." Health Policy & Planning 35(6): 735-751
12. K. Petersen, R. Feldt, S. Mujtaba, M. Mattsson, Systematic mapping studies in software engineering, in: Presented at the 12th International Conference on Evaluation and Assessment in Software Engineering (EASE), 2008. (http://robertfeldt.net/publications/petersen_ease08_sysmap_studies_in_se.pdf)
13. Miki, A. J., et al. (2020). "Using Evidence Mapping to Examine Motivations for Following Plant-Based Diets." Current Developments in Nutrition 4(3): nzaa013.
14. . Althuis, M. D. and D. L. Weed (2013). "Evidence mapping: methodologic foundations and application to intervention and observational research on sugar-sweetened beverages and health outcomes." American Journal of Clinical Nutrition 98(3): 755-768
15. Welsh, E. J., et al. (2015). "Interventions for bronchiectasis: an overview of Cochrane systematic reviews." Cochrane Database of Systematic Reviews(7): CD010337.
16. Sopjani, L., et al. (2020) "Unlocking the Linear Lock-In: Mapping Research on Barriers to Transition." Sustainability 12(3)
17. . Haddaway, N. R., et al. (2017). "How does tillage intensity affect soil organic carbon? A systematic review." Environmental Evidence 6(1).
18. Miake-Lye, I.M., Hempel, S., Shanman, R. et al. (2016) What is an evidence map? A systematic review of published evidence maps and their definitions, methods, and products. Syst Rev 5, 28. https://doi.org/10.1186/s13643-016-0204-x
