# Supplementary material for: What are the methodological characteristics of evidence and gap maps? A systematic review and evidence and gap map
Source: Cochrane Evid Synth Methods. 2024 Aug 5;2(8):e12096. doi: 10.1002/cesm.12096 (PMC11795909; doi:10.1002/cesm.12096)
Supplement: Supplementary file 2 — Supporting information. [file CESM-2-e12096-s002.pdf]

Appendix B copy of data extraction plan/code sets to extract

Data extraction plan

Guiding objective

overarching question: 'what are the characteristics of the methods used to produce, present and update Evidence and Gap Maps (EGM) which impact the utility and usability of EGMs for stakeholders and/or end users.

An evidence and gap map: is defined as Saran and White (2018) as '...a systematic [visual] presentation of the availability of relevant evidence [of effects] for a particular policy domain'4

Extracted data coding areas and child codes

Bibliographic data

- Form of publication
    - Journal article
    - Grey literature
    - Dissertation/thesis
    - Published report
    - Other

- Area of Research
    - Health
    - Environmental
    - Agriculture (and food)
    - Sustainable development
    - Education
    - digital finance
    - Security and justice
    - Software engineering
    - Homelessness
- Year of include
    - 2011
    - 2012
    - 2013
    - 2014
    - 2015
    - 2016
    - 2017
    - 2018
    - 2019
    - 2020
    - 2021
- Citation
- Title
- Title add
- Authors
- Funding
    - Government or government related
    - NGO/charity
    - Multilateral agency (WHO/world bank)
    - Academic/research institution
    - Other

Appendix B copy of data extraction plan/code sets to extract

  - Unclear
  - /not stated

Study details /Attributes of the study

- Topic of interest/ Areas of Research
  - Health
  - Environmental
  - Agriculture (and food)
  - Sustainable development
  - Education
  - digital finance
  - Security and justice
  - Software engineering
  - Homelessness
  - Other
- Population of interest
  - Children (0-15)
  - Young people (16-34)
  - Other Working age people (35-64)
  - Older people (65+)
  - Unclear/not stated
  - Not relevant
- Rationale for the study/map
- Purpose/aim of the study [this may impact how things are mapped]

Codes for study Objectives

- THE MAP DESIGNS
  - Bubble
  - Matrix
  - Matrix like: Grid
  - Matrix like: Table
  - Heatmap
  - Flow diagram (other)
  - Bar chart (other)
  - Effect design plot (other)
  - Tree map (other)
  - Hierarchy list (other)
  - Geographical (other)
- METHODS USED
  - Systematic review
  - Scoping review
  - Systematic map
  - Some form of evidence mapping
  - Umbrella map
- TOOLS, SOFTWARE & PLATFORMS - FOR INTERACTIVE MAPS
  - Eppi- mapper

Appendix B copy of data extraction plan/code sets to extract

  - 3ie maps
  - Tableau
  - HAWC
  - Evi-glance
  - Evi-atlas
  - DCIS evidence map tool
  - Nature and people portal
  - Aero Data Lab
  - Mangotree
  - Transtria
  - Acer map
  - Evidencesynthesisibd.com
  - Not clear
- AREAS STAKEHOLDERS ARE INVOLVED IN
  - Preliminary stage(s)
  - Map format
  - Searching
  - Data processing/synthesis
  - Dissemination activities
  - Not specified
- Maps made with stakeholders
  - YES
  - NO
- **Stakeholder** information NOT FOR FILTER
  - Is there stakeholder engagement
    - YES
    - No
  - HOW were they involved
    - Preliminary stage(s)
      - initial consultation
      - gaining funding
      - topic selection
      - protocol
      - review questions
      - Other BEFORE
    - Map format
      - scope of the map
      - conceptual framework/theory of change
      - Map framework development
      - brainstorming interventions
      - Map visualisation
      - Map codes
      - map validation
    - Searching
      - Search strategy
      - search string
      - search terms
      - request for potential includes
      - Screening (including criteria for)
      - inclusion/exclusion criteria
    - Data process/synthesis
      - Coding (for data extraction)
      - Data extraction
      - data analysis
      - topic groupings (data synthesis)
      - feedback on the map AFTER N>B NOT ASSIGNED TO A 2 GEN CODE YET

        - Dissemination activities
            - reviewing draft report
            - language of the map
            - dissemination of findings
            - OTHER AFTER N.b not assigned to a parent yet *include interpretation of results*
        - not specified
        - Other - During n.b: not assigned to a parent code yet
- GAPS coding will not work in map filter
    - Are gaps mentioned in the background
        - YES
        - NO
    - mentioned in the AIMS (anywhere)
        - YES
        - NO
    - mentioned in the overall aims
        - YES
        - NO
    - mentioned in sub aims/objectives
        - YES
        - NO
    - Mentioned in the methods
        - YES
        - NO
    - mentioned in the results
        - YES
        - No
    - Mentioned in the discussion
        - YES
        - NO
        - if yes then HOW
            - WHAT gaps there are
            - WHERE the gaps are
            - WHY there are gaps (e.g. lack of research funding)
            - IMPACT of the gap
            - OTHER
- Gaps are mentioned:
    - in the study background
    - in the study aims or objectives
    - in the study methods
    - in the study results
    - in the study discussion

Objective f

- plan to update mentioned
    - Yes
    - No
- A living map (mentions being)
    - Yes
    - No
- Map interactivity
    - YES *Map is interactive*
    - NO *Map is not interactive*
    - unsure
- Study Protocol
    - Yes

-
  - No

Codes for map filters

- Map designs for segmenting
  - Bubble
  - Matrix
  - Matrix like
  - Heatmap
  - Other
- STUDY &/or MAP FEATURES
  - Map is interactive
  - Map has a study protocol
  - Map considered a 'Living map'
  - Map had/has a plan to update it

Codes that won't be in the map

- methods used full -.
  - Systematic review
  - Scoping review
  - Systematic map
  - Some form of evidence mapping
    - Evidence mapping
    - making an evidence map
    - Evidence scoping and mapping
    - Mapping review
    - Rapid evidence mapping
    - Evidence review (mapping)
  - Umbrella review
- stakeholder things not for map
  - How - OVER TIME
    - BEFORE
    - DURING
    - AFTER
    - unspecified
    - B & D
    - B & A
    - B & D & A
  - How format and content
    - neither
    - format
    - content
    - not specified
    - unclear
    - only format
    - only content
    - both format and content (not neither)
    - both format and content (and neither)
    - neither and format
    - nether and content
    - only neither
  - count of different ways involved
    - 1
    - 2
    - 3
    - 4
    - 5
    - 6
    - 10
    - not specified
